# Near-Infrared Fluorescence-Enhanced Optical Tomography

**DOI:** 10.1155/2016/5040814

**Published:** 2016-10-10

**Authors:** Banghe Zhu, Anuradha Godavarty

**Affiliations:** ^1^Center for Molecular Imaging, The Brown Foundation Institute of Molecular Medicine, The University of Texas Health Science Center, Houston, TX 77030, USA; ^2^Optical Imaging Laboratory, Department of Biomedical Engineering, Florida International University, Miami, FL 33174, USA

## Abstract

Fluorescence-enhanced optical imaging using near-infrared (NIR) light developed for* in vivo* molecular targeting and reporting of cancer provides promising opportunities for diagnostic imaging. The current state of the art of NIR fluorescence-enhanced optical tomography is reviewed in the context of the principle of fluorescence, the different measurement schemes employed, and the mathematical tools established to tomographically reconstruct the fluorescence optical properties in various tissue domains. Finally, we discuss the recent advances in forward modeling and distributed memory parallel computation to provide robust, accurate, and fast fluorescence-enhanced optical tomography.

## 1. Introduction

Over the past two decades, there has been a considerable interest in the use of near-infrared (NIR) light for deep tissue imaging. Briefly, NIR optical imaging takes advantage of the wavelength range of around 650–900 nm, wherein the major tissue chromophores such as hemoglobin, lipid, and water exhibit their lowest absorption coefficients [1]. Additionally, the interference from tissue autofluorescence is minimized in this wavelength regime, which can further enhance optical imaging contrast [2]. NIR optical imaging is based on the principle of launching NIR light onto the tissue surface and detecting the scattered and attenuated NIR signal. The normal tissues are differentiated from the diseased tissues based on the inherent differences (termed as endogenous contrast) in the optical properties (in terms of absorption and scattering coefficient) of the tissue medium, thus providing physiological information about the tissue. For example, the clinical application of NIR optical imaging technique towards breast cancer diagnosis is based on the intrinsic absorption contrast originating from the tumor angiogenesis and the hypervascularization of tumor periphery [3]. However, the angiogenesis-mediated absorption contrast approaches cannot effectively detect the early cancer and assessment of sentinel lymph node staging, metastatic spread, and multifocality of breast disease [4]. By the use of exogenous NIR fluorochromes and reporter probes, NIR optical imaging technique can overcome these limitations.

Fluorescence-enhanced optical imaging involves the use of fluorescent contrast agents in order to enhance the optical contrast between normal and diseased tissues. In fluorescence-enhanced optical imaging process, when NIR light at the excitation wavelength is launched onto the tissue surface, the photons propagate into the tissues, during which they are minimally absorbed and preferentially scattered. Upon encountering a fluorescent molecule, the photons excite the fluorescent molecules from their ground state to a higher orbital level. After residing at the higher energy orbital for a period defined as the* fluorescence lifetime*, the fluorescent molecule emits fluorescent signal of greater wavelength than the incident NIR light. The quantum efficiency of the fluorescent emission (*ϕ*) is the fraction of excited dye molecules, or activated fluorophores, which relax radiatively. The emitted fluorescent signal along with the perturbed excitation signal propagates in the tissue, before they are detected at the tissue surface. Fluorescence-enhanced optical imaging can potentially offer a high specificity and sensitivity in detecting the early cancer and assessment of sentinel lymph node staging, metastatic spread, and multifocality of breast disease and provide information about the environment of the fluorophore molecules as well as their location by appropriate analysis of reemitted fluorescence signal.

Many fluorescence optical imaging techniques are available for imaging surface (~1 mm) and subsurface (~4 mm) fluorescent events (microscopic and macroscopic imaging modalities with respect to the resulting resolution). The microscopic fluorescence imaging techniques mainly consist of confocal reflectance imaging, multiphoton microscopy, and multiphoton laser scanning microscopy [5]. Owing to the restricted field of view (less than 1 mm in diameter), the microscopic imaging techniques are the most inefficient means to image the small size tissue. Macroscopic fluorescence reflectance imaging (FRI) techniques offer simple photographic methods, in which an array is used for point delivering of laser energy and point collecting of generated fluorescence; or an expanded excitation beam is employed for area illumination and an array detector or an area detector (CCD or CMOS camera) is used for capturing the generated fluorescence on whole small animal or the large size tissue [6, 7]. Appropriate combination of filters is generally introduced to separate the generated fluorescence from strong background excitation light [2, 8]. FRI technique has several limitations, including nonuniformity of the expanded excitation beam, incapability to quantify the fluorochrome, and low imaging quality contaminated by intrinsic light from different tissue layers. Hence, this technique is suitable for imaging of superficial structure and may engender feint if one has not accounted for nonlinear effect dependence on lesion depth and tissue optical properties [9]. In order to resolve and quantify fluorochromes deeper into tissue, tomographic approaches are necessary. This review is focused on the mathematical tools developed towards two-/three-dimensional (2D/3D) fluorescence-enhanced optical tomography.

## 2. Measurement Approaches

**Figure 1:**
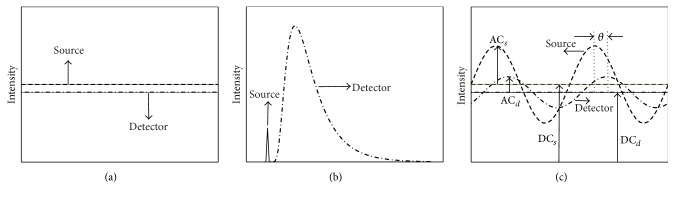
Different measurement approaches in optical imaging: (a) continuous wave, (b) time-domain photon migration, and (c) frequency-domain photon migration.

In general, diffuse (nonfluorescence) or fluorescence-enhanced optical imaging is performed using one of the three measurement approaches: (i) the continuous wave (CW) domain, (ii) the time-domain photon migration (TDPM), and (iii) the frequency-domain photon migration (FDPM) (see Figure 1) [10, 11].

### 2.1. Continuous Wave-Based Measurement Approach

In a CW-based measurement approach, the incident excitation energy from a source (i.e., source intensity) is constant over timescale of milliseconds or modulated at low frequency (a few kHz) and the reemitted fluorescence energy from exogenous agents is likewise constant (see Figure 1(a)). As the excitation light travels through the absorption and scattering medium, it is exponentially attenuated with respect to the incident light. The amount of fluorescence generated from a fluorochrome within the tissue is proportional to the product of the fluorochrome concentration, quantum efficiency, and the local excitation fluence. The propagation of NIR light through tissue is well described by diffusion equation derived from the radiative transport equation [12, 13]. Coupled diffusion equations are employed in order to predict the fluorescence light generation and propagation in tissue, and the equations are given by

(1) ∇·Dxr→∇Φxr→−μaxi+μaxfr→Φxr⃑=−Sxr⃑,∇·Dmr⃑∇Φmr⃑−μami+μamfr⃑Φmr⃑=ϕμaxfr⃑Φxr⃑,

where Φ represents the fluence and *μ*_*a*_ is the absorption coefficient (cm^−1^), where the subscripts *x* and *m* correspond to excitation and emission wavelength, respectively, and the subscripts *i* and *f* denote the chromophores (i.e., the endogenous chromophores in tissues) and fluorophores or exogenous fluorescing agents, respectively; *S*_*x*_ is the excitation photon source; $\vec{r}$ is the positional vector at a given point. The excitation fluence, Φ_*x*_, couples the diffusion equations (1). The optical diffusion coefficients at the excitation wavelength *D*_*x*_ and emission wavelength *D*_*m*_ are given by

(2) $$\begin{array}{c} D_{x,m}\left(\;\vec{r}\;\right)=\frac{1}{3\left[\mu_{ax,m}\left(\vec{r}\right)+\mu_{sx,m}^{\prime }\left(\vec{r}\right)\right]}, \end{array}$$

where *μ*_*s*_′ is the reduced optical scattering coefficient (cm^−1^).

The CW-based measurement approach is relatively simple and requires an inexpensive instrumentation setup. However, this method cannot image the fluorescence decay kinetics (lifetime) nor resolve the scattering and absorption properties of tissue [9]. These limitations can be overcome by using time-dependent measurement approaches (TDPM and FDPM) as described below.

### 2.2. Time-Domain Based Measurement Approach

In a time-domain based measurement approach, ultrafast (with duration range from picosecond to femtosecond) laser pulses are employed to illuminate the tissue and the generated fluorescent signals are detected by a streak camera, time-gated CCD camera, or time-correlated single photon counting device. When a light pulse is launched onto the tissue, its profile will be broadened with nanosecond “time-of-flight” (see Figure 1(b)). The generated fluorescence pulse before being recorded is further broadened owing to the lifetime of the fluorochrome and latterly its propagation inside the scattering tissue. As a result, the recorded fluorescence can be regarded as a function of time at different locations within tissue. In TDPM measured approach, the coupled diffusion equations describing the generation and propagation of a fluorescent wave can be written as [14–17]

(3) ∇·Dx∇Φxr→,t−μaxi+μaxfr→Φxr⃑,t=1cx∂Φxr⃑,t∂t−Sxr⃑,t,1cm∂Φmr−,t∂t−∇·Dmr→∇Φmr−,t+μami+μamfr→Φmr−,t−ϕμaxfr→τ∫0texp⁡−t−t′τΦxr−,t′dt′=0,

where *c*_*x*_ and *c*_*m*_ represent the velocity of light at excitation and emission wavelengths (cm/sec), respectively; *t* and *t*′ denote the photon traveling time (sec) in the tissue and *ϕ* represents the quantum efficiency. The coupled diffusion equations above assume that fluorochrome exhibits first-order single-exponential fluorescent decay kinetics with a constant fluorescence lifetime *τ*. In the case of multiexponential decay kinetics and reabsorption, a similar fluorescence photon density equation can be derived by incorporating the average fluorescence lifetime [18].

In comparison to the CW-based approach, TDPM approach is capable of discriminating the fluorescence decay kinetics from the changes in fluorochrome concentration. On the downside, the signal-to-noise ratio (SNR) of TDPM approach suffers significantly and the cost and complexity of the instrumentation are relatively high [9].

### 2.3. Frequency-Domain Based Measurement Approach

In a frequency-domain based measurement approach, a modulated-intensity light source at radio frequencies ranging from 30 to 200 MHz is employed [2, 19]. FDPM is directly related to TDPM through the Fourier transform. As the intensity-modulated light propagates through the high scattering tissue, it becomes amplitude attenuated and phase-shifted relative to the incident light. Before reaching detectors, the generated fluorescence is further attenuated and phase-shifted owing to the quantum efficiency, lifetime of the fluorochrome, and absorption and scattering properties of the intervening tissue (Figure 1(c)). In FDPM, the coupled diffusion equations for light propagation at a given modulation frequency of light are given by [14–16]

(4) −∇·Dxr⃑∇Φxr→,ω+μaxi+μaxfr→+iωcxΦxr→,ω=Sxr→,−∇·Dmr→∇Φmr→,ω+μami+μamfr→+iωcmΦmr⃑,ω=ϕμaxf11−iωτΦxr⃑,ω,

where *ω* corresponds to the modulation frequency of propagating light. The fluence at excitation and emission wavelength is given by Φ_*x*_ = *I*_AC,*x*_exp⁡(*iθ*_*x*_) and Φ_*m*_ = *I*_AC,*m*_(*iθ*_*m*_), respectively, where *I*_AC_ is the amplitude and *θ* is the phase shift at excitation and emission wavelengths, respectively.

The FDPM-based instrumentation can be operated in a non-light-tight environment due to the fact that the amplitude of the detected fluorescence is insensitive to the ambient light [20]. FDPM-based approach can also discriminate fluorescence decay kinetics (similar to TDPM-based approach). In addition, FDPM approach has an added advantage of considerably high SNR with respect to TDPM approaches, due to steady-state measurements of a time-dependent light propagation process [21, 22]. This approach also retains the signal dependency on fluorescence lifetime (as in TDPM), which is otherwise missing in CW-based approach.

### 2.4. Boundary Conditions

The light propagation models using either of the measurement approaches can be solved by applying appropriate boundary conditions in the finite medium. The three major boundary conditions include (i) the partial current boundary condition, (ii) the extrapolated boundary condition, and (iii) the zero-boundary condition.

#### 2.4.1. Partial Current Boundary Condition

The partial current boundary condition, which is representative of the real physical system, states that the photon leaving the tissue surface never returns, and the Fresnel reflections at the air-tissue interface are determined using a reflection parameter [23]. The boundary condition is given by

(5) $$\begin{array}{c} \Phi_{x,m}\left(\;\vec{r},w;\vec{r},t\;\right)+2\gamma D_{x,m}\left(\;\vec{r}\;\right)\frac{\partial \Phi_{x,m}\left(\vec{r},w;\vec{r},t\right)}{\partial \vec{n}}=0, \end{array}$$

where *γ* is the index-mismatch parameter, which is a function of the effective refractive index at the boundary surface; $\vec{n}$ is the unit surface vector normal to the imaging plane; *ω* and *t* correspond to the frequency and time domain, respectively.

#### 2.4.2. Extrapolated Boundary Condition

The extrapolated boundary condition is a simplified form of the partial current boundary condition [13, 24, 25] and can be implemented by setting the fluence rate to zero at an extrapolated boundary located at a distance, *z*_*b*_, outside the domain:

(6) $$\begin{array}{c} \Phi_{x,m}\left(\;\vec{r},w;\vec{r},t\;\right)=0\;\text{at }z=z_{b}. \end{array}$$

An approximate value for *z*_*b*_ was estimated to include the Fresnel reflection at the surface and is given in terms of the index-mismatch parameter and diffusion coefficient as [26]

(7) $$\begin{array}{c} z_{b}=2\gamma D_{x,m}\left(\;\vec{r}\;\right). \end{array}$$

#### 2.4.3. Zero Fluence Boundary Condition

In the zero fluence boundary condition, the fluence at and outside the boundary is set to zero:

(8) $$\begin{array}{c} \Phi_{x,m}\left(\;\vec{r},w;\vec{r},t\;\right)=0\;\text{at }z=0. \end{array}$$

This is a simpler boundary condition mathematically and it is good approximation for biological tissues, but it does not accurately represent the real physical system [13, 26].

## 3. Mathematical Tools in Fluorescence-Enhanced Optical Tomography

The coupled diffusion equations are used along with one of the boundary conditions above, in order to solve for the parameter of interest. The optical tomography problem is solved in three steps. As a first step, the interior optical property map of the tissue medium is assumed known and the coupled diffusion equations are solved for the fluence at either wavelength (termed as* forward problem*). As a second step, the fluence obtained from the forward model is compared to the acquired boundary surface measurements (experimental or simulated), in order to validate the light propagation model employed for fluorescence-enhanced optical tomography; in other words, model validation is performed on known phantoms. As a third and final step, the acquired boundary surface measurements are used along with the coupled diffusion equations in order to estimate the interior optical property map (termed as* inverse problem*); in other words, inversions are performed assuming that the phantom properties are unknown. In an actual experimental study containing unknown phantoms, the acquired boundary surface measurements are used along with the light propagation model in order to solve the* inverse problem* (i.e., third step) directly. Details of the forward and inverse problem in fluorescence-enhanced optical tomography are described in the following sections.

### 3.1. Forward Problem

In the forward problem of fluorescence-enhanced optical tomography, one may assume that the optical properties of the entire tissue medium are known in order to predict the boundary surface measurements (in either of the three measurement approaches described in earlier sections). The fluence governed by the coupled diffusion equations can be estimated using empirical, analytical, and numerical methods as described in the following subsections.

#### 3.1.1. Empirical Method

In the empirical method, the entire domain is generally discretized into cubic elements (3D) or square elements (2D) and each element corresponds to a weight. Many investigators have utilized model systems to empirically measure the weights. For instance, Fantini et al. [27] have studied the variations of the measured signals when a small point-like absorbing target was introduced into an otherwise homogenous medium. By moving the small target to each element, a set of weights for a particular source-detector pair was generated empirically. In the case of a semi-infinite medium, this set of weights takes up the shape of a banana function. The multiplication of the weights with the optical properties (assumed known) of the entire tissue medium in turn provides the fluence values. To date, empirical methods have not been implemented for fluorescence-enhanced optical tomography studies. However, these methods provide more realistic predication of the fluence and have potential for their application in fluorescence-enhanced optical tomography studies.

#### 3.1.2. Analytical Method

In CW and FDPM domains, the coupled diffusion equations can be reduced to their related Helmholtz equations by making suitable assumptions and approximations, such as the Born or Rytov approximation [28]. Using Green's function theorem, one can easily obtain an integral expression for the emission fluence:

(9) Φmr→s,r→d=∫ΩGfr→d,r→′ϕμaxfr→′Dmr→1−iωτΦxr→′,r→sdΩ,

where *Ω* is the volume of integration, ${\vec{r}}_{d}$ and ${\vec{r}}_{s}$ are the location of point detector and source, respectively, and ${\vec{r}}^{\prime }$ is the point location in the region of interest. For an infinite geometry, Green's function is $G_{f}\left({\vec{r}}_{d},{\vec{r}}^{\prime }\right)=\exp\left(ik_{m}\left|{\overset{⃑}{r}}_{d}-{\vec{r}}^{\prime }\right|\right)/4\pi \left|{\vec{r}}_{d}-{\vec{r}}^{\prime }\right|$, where *k*_*m*_ is the wave number. For the regular boundaries, such as slab or semi-infinite geometry, Green's function can also be derived analytically by using an angular spectrum algorithm [29] or a plane-wave expansion [30]. Although empirical and analytical methods are direct and fast, they are applicable for regular boundaries. For arbitrary boundary shapes, it is difficult to incorporate these irregular shapes into the solution of the coupled equations analytically, and hence numerical methods (such as the finite difference method, the finite element method, or the boundary element method) are employed at the cost of computation speed.

#### 3.1.3. Numerical Methods

*(1) Finite Difference Method*. In the finite difference method (FDM), the entire domain is discretized into square (2D) or cubic (3D) elements, respectively, and each node of every element is assumed a known parameter. The mesh is finely resolved in order to minimize the discretization error at the cost of increasing dimensionality of the problem. Hence, the forward problem becomes computationally intense in the case of large 3D domain. This problem can be overcome by using multigrid finite difference methods over single grid method [31–34]. In the multigrid FDM, several sizes of gird are employed simultaneously, such as using a coarse grid to provide an initial guess to the solution on successive finer grids. The process is continued until the desired resolution is reached. Not only is the multigrid method faster than the single grid method, but also the method reduces the discretization errors, while maintaining the resolution of the reconstructed image [31, 35].

*(2) Finite Element Method.* The finite element method (FEM) [36–38] is suitable for any geometry involving the discretization of the entire domain into triangle elements (2D) or tetrahedral, pyramidal, and hexahedral elements (3D). However, unlike the finite difference methods, FEM can be employed on curvilinear domains, such as the physiological tissue shapes, which minimizes the discretization errors and reduces the computational time in the inverse problem upon appropriate coding [36–39]. Typically, the finite element method is formulated using the Galerkin approximation, where the second-order coupled diffusion equations are converted to first-order differential equations. The solutions of these first-order differential equations are in turn approximated as a linear function in space within each finite element. The challenges are in generating a finite element mesh for an irregular object with complex internal structure and developing a robust, efficient 3D meshing technique. An adaptive finite element method has been proposed, in which the maps of the forward/adjoint variables and the unknown parameters are discretized separately in adaptively refined meshes, enabling computationally efficiency during tomographic reconstructions [40, 41].

In both the finite difference and finite element method, discretization of the mesh plays a significant role in minimizing model mismatch errors (difference between experimental measurements and predicted measurements obtained from the forward model) and eventually impacting the quality and accuracy of image reconstructions.

*(3) Boundary Element Method*. In the boundary element method (BEM), the entire domain is divided into a finite number of spatially coherent 3D regions, each of which can be regarded as homogeneous. One only needs to discretize the boundaries of these subdomains into nodes and 2D elements. Imposing the constraints of compatibility and equilibrium on shared boundaries between subdomains, one can employ analytical solutions inside each subdomain. In comparison to FEM, BEM requires significantly fewer nodes and elements and is subject to less discretization error. In experimental fluorescence-enhanced optical imaging studies, BEM gave more accurate and stable solutions of the excitation and emission equations (i.e., forward problem) in comparison to the solutions using FEM [42, 43]. The forward problem offers a unique solution of the coupled diffusion equations. By employing the forward model of the coupled diffusion equation and the experimentally measured data on boundary surface, we can solve the inverse problem, giving rise to 3D tomographic reconstructions.

### 3.2. Inversion Problem

Unlike the forward problem, the inverse problem of fluorescence-enhanced optical tomography is a complicated problem to be solved. Herein, sparse boundary surface measurements obtained experimentally for 3D tissue phantom domain are used to reconstruct the unknown parameters or optical properties at every point of the entire 3D domain. Typically, the number of unknowns (optical properties) is significantly greater than the total number of boundary surface measurements, and the inverse problem is underdetermined. Hence, the solutions are “ill-posed” which means that the solution is nonunique and unstable, especially in the presence of measurement error that is actually acquired in the measurement set. There are various iterative approaches available to solve the inverse problem in optical tomography, which can mainly be categorized as (i) singular value decomposition method, (ii) algebraic reconstruction technique, (iii) Newton's optimization method, (iv) Bayesian reconstruction techniques, and (v) conjugate gradient method.

#### 3.2.1. Singular Value Decomposition Method

The singular value decomposition (SVD) can be directly derived from the theory of linear algebra. By use of SVD approach, the weight matrix *W* obtained by solving the coupled diffusion equations, using analytical solution or empirical method described above, can be decomposed into three matrixes *U*, *S*, and *V*. The columns of matrix *U* represent the detection-space modes of *W* and are orthogonal, *S* is a diagonal matrix, and the columns of matrix *V* represent the image-space modes of *W* and are orthogonal. Since matrix *W* must be square before performing inversion operation, one first simply pads this matrix with rows of zeros or columns of zeros and then inverses the matrix *W* according to

(10) W=U·diag⁡sj·VT,W−1=VT−1·diag⁡sj−1·U−1,W−1=V·diag⁡1sj·UT.

If the matrix is singular, the corresponding eigenvalue *s*_*j*_ equals zero and 1/*s*_*j*_ can be set to zero. Using a smoothing algorithm, that is, *s*_*j*_ → *s*_*j*_ + *σ*/*s*_*j*_, where herein *σ* is a free parameter and can be optimized empirically, one can improve the quality of image reconstructions. SVD method has been employed for reconstructing the distributions of fluorescing agent in small animals (mice) using CW measurements [44, 45]. The SVD approach involves the computation of the matrix inversion leading to long computing times in case of large 3D tissue geometries/volumes. Therefore, this approach is limited to small tissue geometries, small animals, or in cases where lower-resolution conditions are sufficient.

#### 3.2.2. Algebraic Reconstruction Technique

The algebraic reconstruction technique (ART) and its generations are widely used to solve the linear system of equations. In order to locate the solution, an initial guess of solution is first made in hyperplanes with *N* dimension. This initial guess is projected onto the first line of the hyperplanes. The resulting point on the first line is reprojected onto the second line, and so on, until the *N*th line. These *N*'s movements constitute one iteration, then projecting back onto the first line and so forth. If there exists a unique solution, the iteration will always converge to that point. The prediction of emission fluence Φ_*m*_ is as follows:

(11) $$\begin{array}{c} \Phi_{m}=WX, \end{array}$$

where *X* denotes unknown optical properties and the unknown parameters can be updated as follows:

(12) $$\begin{array}{c} X_{k+1}^{j}=X_{k}^{j}+\eta \cdot \frac{\Phi_{\exp t}-\sum_{l=1}^{N_{mesh}}W_{kl}X_{k}^{l}}{\sum_{l=1}^{N_{mesh}}{\left|W_{kl}\right|}^{2}}\cdot W_{kj}, \end{array}$$

where *l*, *j* = 1,…, *N*_mesh_ and *N*_mesh_ represents the total number of elements in the 2D and 3D domain of interest; *k* = 1,…, *N*_source_ · *N*_detector_ · *N*_iteration_; *N*_source_ and *N*_detector_ are the number of sources and detectors, respectively; *N*_iteration_ is the number of iterations; Φ_exp⁡*t*_ represents the experimental fluence. The relaxation parameter *η* is introduced in order to reduce the effect of noise in ART reconstruction and this parameter can be made as a function of iteration number. The iterative procedure continues as a loop, until convergence is obtained. A simultaneous iterative reconstruction technique (SIRT) involves moving the starting point to the *N* lines, respectively, and the obtained *N* solutions are averaged as a new input. SIRT offers an improved image quality in comparison to the images obtained using the ART but at the expense of a relatively slow convergence.

The ART and SIRT have been widely employed in fluorescence-enhanced optical tomography studies [39, 46–53]. Intes et al. [54] proposed a method to enhance convergence rate by selecting appropriate projection access order in ART. In comparison to the SVD method, the ART method allows imposition of hard constraints on the reconstructed optical parameters (e.g., absorption coefficient can be set to zero for a negative value) and hence greatly improves the quality of image reconstructions.

#### 3.2.3. Newton's Optimization Approaches

The inverse problem can be solved by the method of least squares. Here, we define the error function as the sum of square of errors between the measured Φ_exp⁡*t*_^*i*^ and the calculated Φ_*m*_^*i*^ value of fluence, at detector *i* = 1,…, *M*:

(13) $$\begin{array}{c} F\left(\;X\;\right)=\overset{M}{\underset{i=1}{\sum }}{\left[\;\Phi_{\exp t}^{i}-\Phi_{m}^{i}\;\right]}^{2}=\overset{M}{\underset{i=1}{\sum }}{\left[\;f_{i}\left(\;X\;\right)\;\right]}^{2}, \end{array}$$

where *M* = *N*_source_ *∗N*_detector_ (i.e., total number of source-detector pairs) and *f*_*i*_ refers to a residual of the difference between the measured value and the calculated value. The gradients of the error function with respect to the property, *X*, and Taylor's expansion of function *F* around a small perturbation of optical property, Δ*X*, yield the function *Y*(Δ*X*), which is minimized:

(14) YΔXFX+ΔX−FX=2JTfX·ΔX+2·ΔXTJTJ+∑i=1MfiX∇2fiX·ΔX,

where *J* is a Jacobian matrix, given by ∂(ΔΦ_*i*_)/∂*X*_*j*_. If the second term on the right-hand side of (14) is neglected, the equation represents first-order Newton's method and its minimization leads to Gauss-Newton's method:

(15) ∇YΔX⟹0=JTJ·ΔX+JTfX,JTJ·ΔX=−JTfX.

In first-order Newton's method and Gauss-Newton's method, the solution is not stable. To stabilize the solution of the inverse problem and make it more tolerant to measurement error, one of the following optimization approaches is typically used.

*(i) Levenberg-Marquardt Algorithm*. Regularization approaches play an important role in the development of algorithms, such as Levenberg-Marquardt algorithm. By introducing a regularization parameter *λ* in Gauss-Newton's method, the Levenberg-Marquardt algorithm of optimization becomes

(16) $$\begin{array}{c} \left[\;J^{T}J+\lambda I\;\right]\cdot \Delta X=-J^{T}f\left(\;X\;\right). \end{array}$$

The choice of the regularization parameter is generally arbitrary or through* a priori *information. Regularization results in a more stable solution to the inverse problem and also improved tolerance to measurement error. The Levenberg-Marquardt algorithm performs poorly in a large residual problem and hence is limited to a small residual problem. Truncated Newton's method was proposed in order to overcome this limitation.

*(ii) Gradient-Based Truncated Newton's Method*. Roy and Sevick-Muraca [55] developed a gradient-based truncated Newton's method by retaining the second term on the right-hand side of (14) and setting the gradient of function *Y*(Δ*X*) to zeros. The equation can be written as

(17) $$\begin{array}{c} \left[\;J^{T}J+\overset{M}{\underset{i=1}{\sum }}f_{i}\left(\;X\;\right)\nabla^{2}f_{i}\left(\;X\;\right)\;\right]\cdot \Delta X=-J^{T}f\left(\;X\;\right). \end{array}$$

For the large residual problem, truncated Newton's method is more robust than Gauss-Newton's and the Levenberg-Marquardt algorithms. This method has demonstrated the feasibility to reconstruct fluorescence lifetime and absorption coefficient in 3D and slab geometries, using simulated data (containing noise, such that it mimics experimental data) [56]. Roy et al. also proposed gradient-based truncated Newton's method along with the penalty/modified barrier function to minimize the objective function for the large-scale problem, called PMBF/CONTN (penalty barrier function with simple bounds constrained), and this method has been demonstrated for fluorescence-enhanced FDPM tomography [57, 58].

*(iii) Active Constrained Truncated Newton Method*. Following truncated Newton's method, Roy and Sevick-Muraca developed an active constrained truncated Newton's method for simple-bound optical tomography, which requires less computational time and storage resource [59]. Based on the physics of the problem, the recovered parameter of fluorescent optical properties (e.g., absorption coefficient or fluorescence lifetime) in fluorescence-enhanced optical tomography must be positive. In the first iteration, the optical property map is recovered and the parameter estimates will be plus and minus a small bounding parameter, if they lie between an upper and lower bounds. The estimated parameter in first iteration severs as an input for next iteration, and the process continues until convergence is reached. The resolution and the performance of tomographic imaging depend on the bounding parameter. Simulated studies verified that active constrained truncated Newton's method may offer a more logical approach than unconstrained optimization for reconstruction of fluorescence optical properties on large 3D tissue phantom containing contrast agents [59].

#### 3.2.4. Bayesian Reconstruction Techniques

Eppstein and coworkers proposed a novel Bayesian reconstruction technique, called the Approximate Extended Kalman Filter (AEKF) algorithm, by using actual measurement error statistics to govern the choice of varying regularization parameters [34, 60]. Here, Newton's solution is formulated as

(18) ΔX=JTQ+R−1J+Pxx−1−1·JTQ+R−1·fX,

where *Q* represents the system noise covariance resulting from the inherent model mismatch between the forward model and actual physics of the problem; *R* denotes the covariance of the measurement error; and *P*_*xx*_ is the recursively updated error covariance of the unknown parameters *X*, which is estimated from the measurement error, *f*(*X*). In 3D fluorescence-enhanced optical tomography, the AEKF approach has been employed for reconstruction of the fluorescence absorption coefficients [19, 60–66] and fluorescence lifetime [67] using FDPM-based measurements.

An APPRIZE (Automatic Progressive Parameter-Reducing Inverse Zonation and Estimation) algorithm is a combination of the AEKF and [68] and a data-driven zonation (DDZ) technique, which is used for accelerating the convergence. By using cluster analysis and random field union, the spatially adjacent voxels with the similarly updated estimates are merged into larger stochastic parameter “zones.” Thus, the number of unknown parameters, *X*, decreases in a data-driven fashion. This APPRIZE algorithm has been used for 3D tomographic reconstruction studies in simulated and experimental slab phantoms, demonstrating the effectiveness of DDZ [61, 68]. Compared to the traditional Newton iterative method, the AEKF method and its combination with DDZ technique are more accurate and orders of magnitude faster way.

#### 3.2.5. Conjugate Gradient Techniques

A Newton-like method poses an insurmountable computational burden as the dimension of problem region becomes large. Therefore, it is reasonable to consider gradient-based algorithms, such as conjugate gradient descent (CGD) [69]. Here, the objective function Ψ is defined as

(19) $$\begin{array}{c} \Psi =\frac{1}{2}\overset{N_{\text{source}}}{\underset{i=1}{\sum }}\;\overset{N_{\text{detector}}}{\underset{j=1}{\sum }}{\left(\;\frac{\Phi_{\exp t}^{i,j}-\Phi_{m}^{i,j}}{\sigma_{i,j}}\;\right)}^{2} \end{array}$$

resulting in a total number of measurements *M* = *N*_source_ × *N*_detector_. Equation (19) can be denoted in vector form as

(20) $$\begin{array}{c} \Psi =\frac{1}{2}{\left(\;{\vec{\Phi }}_{\exp t}-{\vec{\Phi }}_{m}\;\right)}^{T}R^{-2}\left(\;{\vec{\Phi }}_{\exp t}-{\vec{\Phi }}_{m}\;\right)=\frac{1}{2}{\vec{b}}^{T}\vec{b}, \end{array}$$

where Φ_exp⁡*t*_^*i*,*j*^ corresponds to the *j*th experimental measurement from *i*th source with standard derivation, *σ*_*i*,*j*_; *b*_*i*,*j*_ = *σ*_*i*,*j*_^−1^(Φ_exp⁡*t*_^*i*,*j*^ − Φ_*m*_^*i*,*j*^) is the residual data for this source-detector pair (*i*, *j*); and *R* is the data-space correlation matrix having the following form:

(21) $$\begin{array}{c} R=\mathrm{diag}\left(\;\sigma_{1,1},\sigma_{1,2},\ldots ,\sigma_{N_{source},1},\ldots ,\sigma_{N_{source},N_{detector}}\;\right). \end{array}$$

In order to solve the optimization problem, the *k*th component of the objective function's gradient is written as

(22) $$\begin{array}{c} \frac{\partial \Psi }{\partial x_{k}}=\overset{N_{source}}{\underset{i=1}{\sum }}\;\overset{N_{detector}}{\underset{j=1}{\sum }}\left(\;\frac{\Phi_{\exp t}^{i,j}-\Phi_{m}^{i,j}}{\sigma_{i,j}^{2}}\;\right)\left(\;\frac{\partial \Phi_{m}^{i,j}}{\partial x_{k}}\;\right) \end{array}$$

whose vector form is

(23) $$\begin{array}{c} \vec{V}=-\overset{M}{\underset{i=1}{\sum }}{J_{i}}^{T}{\vec{b}}_{j}=-J^{T}\vec{b}, \end{array}$$

where Jacobian matrix, *J*, has the size of *M* × *N*_*T*_ and *N*_*T*_ is the number of unknown coefficients of the optical properties. In order to find the minimum of the objective function, that is, ∂Ψ/∂*x*_*k*_, a set of conjugate search directions is generated and a one-dimensional line minimization along the current search direction is performed at each iteration step. CGD method has been employed for 2D/3D studies on phantoms [70–73]. In comparison to the Newton-type method, the gradient method only needs to compute the gradient $\vec{V}$ according to (23), avoiding the construction and inversion.

## 4. Conclusions

This review is limited to the frequently utilized approaches of the coupled diffusion equations in modeling the excitation and emission light propagation in tissues and the absorption of fluorescent agents (not fluorescence lifetime due to the fact that there are few contrast agents designed with “tuneable” lifetimes) in the inversion imaging. The diffusion equation is an approximation of the radiative transfer equation (RTE) despite its known inaccuracy in high absorption domains [74–77]. Large reconstruction localization errors and artifacts from diffusion equation-based reconstruction significantly affect the acquisition of quantitative biological information [78, 79]. There have been a few attempts to use the RTE and/or its high-order approximations but with low implementation efficiencies [80–82]. Because of the tremendous computation dimension in the RTE simulation, distributed memory parallel computation is needed. Traditional solutions have included central processing unit (CPU) based moderately parallel system with shared memory access (multiprocessor and multicore implementation). However, the large-scale distributed parallel systems are limited by data transformer between nodes. More recently, the parallel architecture of graphics processing units (GPU) has been utilized for the acceleration of general purpose computations for the solution of sparse linear system [83]. With these advances in improving computation efficiency, more accurate and fast fluorescence-enhanced optical tomography will become possible, and this will accelerate its clinical translation.
